# A Case for the Non-Neurologist Telestroke Provider

**DOI:** 10.3389/fneur.2021.651519

**Published:** 2021-08-06

**Authors:** Justin Choi, Ashley Petrone, Amelia Adcock

**Affiliations:** ^1^School of Medicine, West Virginia University, Morgantown, WV, United States; ^2^Department of Neurology, West Virginia University Hospitals, Morgantown, WV, United States; ^3^Department of Neurology, Mayo Clinic, Phoenix, AZ, United States

**Keywords:** telestroke, telemedicine, stroke, telestroke program, emergency & critical care

## Abstract

**Introduction:** Telestroke networks have effectively increased the number of ischemic stroke patients who have access to acute stroke therapy. However, the availability of a dedicated group of stroke subspecialists is not always feasible. We hypothesize that rates of tPA recommendation, sensitivity of final diagnosis, and post-tPA hemorrhagic complications do not differ significantly between neurologists and an emergency-medicine physician during telestroke consultations.

**Methods:** Retrospective review of all telestroke consults performed at a comprehensive stroke center over 1 year. Statistical analysis: Chi squared test.

**Results:** Three hundred and three consults were performed among 6 spoke sites. 16% (48/303) were completed by the emergency medicine physician; 25% (76/303) were performed by non-stroke-trained neurologists, and 59% (179/303) were completed by a board-certified Vascular Neurologist. Overall rate of tPA recommendation was 40% (104/255), 38% (18/48), 41% (73/179), and 41% (31/76) among the all neurology-trained, emergency medicine-trained, stroke neurology-trained and other neurology- trained provider groups, respectively (*p* = 0.427). Sensitivity of final stroke diagnosis was 77% (14/18) and 72% (75/104) in the emergency-medicine trained and neurology-trained provider groups (*p* = 0.777) No symptomatic hemorrhagic complications following the administration of tPA *via* telestroke consultation occurred in any group over this time period. One asymptomatic intracerebral hemorrhage was observed (0.96% or 1/104) in the neurology-trained provider group.

**Discussion/Conclusion:** Our results did not illustrate any statistically significant difference between care provided by an emergency medicine-trained physician and neurologists during telestroke consultation. While our study is limited by its relatively low numbers, it suggests that identifying a non-neurologist provider who has requisite clinical experience with acute stroke patients can safely and appropriately provide telestroke consultation. The lack of formerly trained neurologists, therefore, may not need to serve as an impediment to building an effective telestroke network. Future efforts should be focused on illuminating all strategies that facilitate sustainable telestroke implementation.

## Introduction

Stroke remains a leading cause of death and disability in the United States, especially in rural, geographically isolated areas (1, 2). Although outcome-changing therapies such as thrombolysis with tissue plasminogen factor (tPA) and endovascular thrombectomy (EVT) are available, these interventions are time-sensitive and limited by patients' proximity to adequately equipped centers (3). Telestroke, usually with neurologist evaluation, has been a critical tool used to make these therapies accessible to patients despite being far from stroke centers. While advances in technology have markedly increased access to audiovisual communication, access to specialists with neurologic expertise continue to be in short supply nationally with insufficient numbers of graduating neurologists only projected to worsen by 2025 (4). However, considering the algorithmic nature of emergency stroke management, how essential is subspecialty care in telestroke evaluation? No formal requirements for the practice of telestroke currently exist, however, the American Telemedicine Association recently advocated that consults be performed by vascular neurologists (5). Previous research has focused on demonstrating the reliability of delivering neurologic care *via* telemedicine as opposed to evaluating the reliability of non-stroke practitioners delivering that care (6).

To bridge the gap between the supply of neurologists and the demand for stroke care, we included an emergency medicine-trained physician in our pool of telestroke providers. The purpose of this article is to determine if a non-neurologist provider with adequate clinical experience with acute stroke can safely and effectively provide telestroke consultation without subspecialist intervention.

## Materials and Methods

This study was approved by the Institutional Review Board at West Virginia University (WVU). Written informed consent was waived and therefore was not required for this study in accordance with national legislation and institutional requirements. A retrospective review of all telestroke consults performed over a 1-year period by our comprehensive stroke center across all six spoke sites was conducted. Spoke sites initiate consults by directly calling the on-call telestroke provider. Following a brief discussion of the relevant clinical details and a review of the patient's computed tomography (CT) scan *via* a cloud-based radiology platform, the telestroke physician conducts an audio-visual consult using a mobile-based encrypted platform with the help of the originating site provider or nurse at bedside. Recommendations regarding patient treatment and disposition are then made. Each formal telestroke evaluation is documented in the hub's electronic medical record. Basic information is collected, including demographic data, location of call, providers involved, relevant time metrics, provisional diagnosis, administration of thrombolytic, or transfer for EVT. Patients are subsequently entered into an internally managed database where outcome information is recorded. Outcomes are obtained *via* the electronic medical record or from information obtained directly from the originating sites during periodic data collection timepoints mandated in the telestroke service contract. This entire process is managed by the telestroke program's coordinator and medical director.

Consults were categorized by three board-certified physician groups: (1) Vascular Neurology, (2) Neurology, or (3) Emergency Medicine. The frequency of tPA use, any radiographic, or clinical evidence of hemorrhage following lytics and sensitivity of initial stroke diagnosis were recorded. Symptomatic intracranial hemorrhage was defined as any acute intracranial hemorrhage on follow-up imaging accompanied by neurologic worsening as defined by an increase of ≥ 4 points on the National Institutes of Health Stroke Scale (NIHSS). Sensitivity of stroke diagnosis was determined based on final ischemic stroke diagnosis at discharge as documented in the medical record. The authors (AA, JC who abstracted this data) were blinded to the identity of the telestroke physician initially involved in the case. Following abstraction, author AP analyzed the results and correlated with the telestroke provider group. In addition to descriptive statistics, we used the Fisher's exact test to calculate *p*-values for any statistically significant findings.

## Results

Three hundred and three telestroke consults were performed over six spoke sites during the study period. 59% (179) of consults were completed by board-certified vascular neurologists, 25% (76) of consults were completed by neurologists, and 16% (48) of consults were completed by an emergency medicine physician. The overall rate of tPA administration was similar between all three provider groups; 41% (73), 41% (31), and 38% (18) in the vascular neurologist, neurologist, and emergency medicine groups, respectively (*P* = 0.427). The sensitivity of final ischemic stroke diagnosis among those who received tPA was also similar between the neurology-trained and emergency medicine-trained provider groups; 72% (75), and 77% (14), respectively (*P* = 0.777). No symptomatic or fatal hemorrhages were observed among patients administered tPA by any of the groups. One non-fatal, asymptomatic intracerebral hemorrhage was observed among the patients administered tPA by the neurology-trained provider group. No radiographic or clinical evidence of hemorrhage occurred among patients given tPA by the emergency medicine-trained provider group.

## Discussion/Conclusion

The rate of tPA in acute ischemic stroke has nearly doubled from the early 2000's and telestroke has been a considerable basis for its expanded use (7, 8). However, a rural-urban disparity persists with urban stroke patients eligible for thrombolysis at least twice as likely to receive it as compared to their rural counterparts (2). Although, recent rural telestroke networks have demonstrated significant impact in tPA use, the evaluating telestroke physician has traditionally been a neurologist (5, 9). In fact, lack of access to a neurologist has been identified as a significant barrier to the use of tPA in ischemic stroke, initially setting the stage for telestroke network creation (10). However, there are currently only 60 neurologists practicing in our state, approximately half of the 145 estimated by the American Academy of Neurology needed to adequately serve this population by 2025 (4, 11). The demand for neurologists already exceeds supply in most states, and this is only projected to worsen by 2025 in the face of the aging US population and the increasing utilization of neurologic services (4). In the face of this growing demand in an increasingly neurologist-strapped environment, we must design a system of care that allocates our limited resources wisely while still delivering safe and effective care to isolated, rural populations far from stroke centers. The benefit of advanced nurse practitioners with subspecialty expertise is well-established and similar models in vascular neurology have been identified but large-scale adoption is lagging (12).

Mobile Stroke Units (MSU) are ambulances equipped with a CT scanner (and in some cases can also perform advanced CT angiography and perfusion) outfitted for thrombolysis administration. In efforts to increase the efficiency of acute stroke treatment, this model brings tPA to the stroke patient in the field. MSU implementation studies have repeatedly demonstrated both feasibility and a reduction in time to treatment (13). They also may be cost effective, at least in densely populated urban settings where multiple tertiary stroke care centers are available (14–16). Their role in rural and resource-limited settings, however, has not been systematically evaluated (17). Furthermore, MSUs are still dependent on a *neurologist* present and staffing these stroke calls in the ambulance. Given these inherent challenges, they are unlikely to be widely adopted in rural settings where effective telestroke and pre-hospital triage systems have been demonstrated to be more impactful (17).

Our results failed to demonstrate statistically significant differences in tPA administration rates, sensitivity of ischemic stroke definitive diagnosis, and hemorrhagic complications between care provided by neurologists and care provided by an emergency medicine physician during telestroke consultation. This suggests that, with adequate clinical experience in management of acute stroke patients and familiarity with thrombolytic inclusion and exclusion criteria, non-neurologist providers can provide safe, and equally effective consultation *via* telestroke. The lack of formerly trained neurologists, therefore, may not necessarily be a fixed barrier to building an effective telestroke network going forward.

It is important to note the several limitations of this study, including its retrospective nature which introduces a risk of selection bias. Also, only one non-neurologist provider was included on the telestroke team for this study, and their training in emergency medicine, a field with significant experience and training in the diagnosis and management of acute ischemic stroke, may have contributed to the similarities between provider groups. Our results, therefore, cannot serve as definitive evidence supporting the broader application of non-neurologists providing telestroke services; rather, they challenge us to seek innovative solutions for the common yet critical clinical field of acute stroke care.

## Data Availability Statement

The original contributions presented in the study are included in the article/supplementary material, further inquiries can be directed to the corresponding author/s.

## Ethics Statement

Ethical review and approval was not required for the study on human participants in accordance with the local legislation and institutional requirements. Written informed consent for participation was not required for this study in accordance with the national legislation and the institutional requirements.

## Author Contributions

JC made substantial contributions to analyzing and interpreting data for the work, drafting the work, and revising the work to be published. AA made substantial contributions to the conception and design of the work, the acquisition and analysis of data for the work, and the drafting and revision of the work to be published. AP made substantial contributions to the acquisition and interpretation of data for the work, and the revision of the work to be published. All authors contributed to the article and approved the submitted version.

## Conflict of Interest

The authors declare that the research was conducted in the absence of any commercial or financial relationships that could be construed as a potential conflict of interest.

## Publisher's Note

All claims expressed in this article are solely those of the authors and do not necessarily represent those of their affiliated organizations, or those of the publisher, the editors and the reviewers. Any product that may be evaluated in this article, or claim that may be made by its manufacturer, is not guaranteed or endorsed by the publisher.
